# Crystal structure of 3-[4-(benz­yloxy)phen­yl]-2,3-di­hydro-1*H*-benzo[*f*]chromen-1-one

**DOI:** 10.1107/S1600536814020868

**Published:** 2014-09-20

**Authors:** R. Vasanthi, D. Reuben Jonathan, K. S. Ezhilarasi, S. Sathya, G. Usha

**Affiliations:** a PG and Research Department of Physics, Queen Mary’s College, Chennai-4, Tamilnadu, India; b Department of Chemistry, Madras Christian College, Chennai-59, India

**Keywords:** crystal structure, flavone, chalcone, chromenone, C—H⋯O hydrogen bonding

## Abstract

In the title compound, C_26_H_20_O_3_, the pyran ring has a distorted half-chair conformation and its mean plane is inclined to the naphthalene ring system, to which it is fused, by 10.79 (9)°. The dihedral angles between the napthalene unit and the benzene and phenyl rings are 54.39 (9) and 52.65 (12)°, respectively, while the benzene and phenyl rings are inclined to one another by 74.80 (14)°. There is a short C—H⋯O contact in the chromen-1-one unit. In the crystal, mol­ecules are linked by two pairs of C—H⋯O hydrogen bonds, forming inversion dimers described by graph set motifs *R*_2_^2^(8) and *R*_2_^2^(10), giving rise to chains running parallel to (101). The chains are linked *via* C—H⋯π inter­actions, forming sheets lying parallel to (010).

## Related literature   

For the biological activity of flavone derivatives, see: Thomas *et al.* (2013 ▶); Kumar *et al.* (2014 ▶); Lee *et al.* (2014 ▶). For the synthesis of the title compound, see: Kumar *et al.* (2014 ▶).
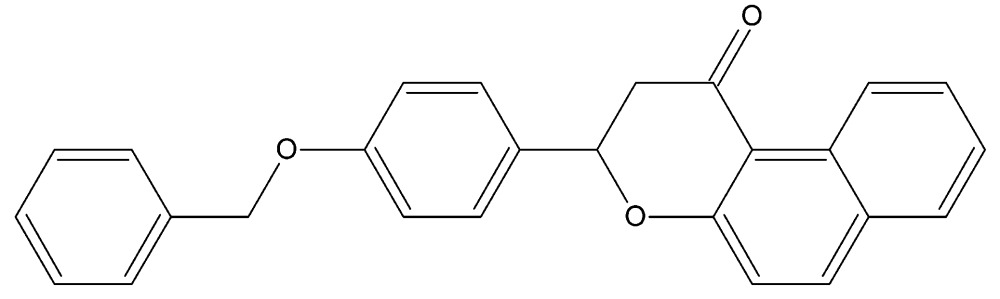


## Experimental   

### Crystal data  

C_26_H_19_O_3_
*M* *_r_* = 379.41
Monoclinic, 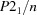

*a* = 6.9632 (4) Å
*b* = 35.846 (2) Å
*c* = 7.7879 (5) Å
β = 100.375 (3)°
*V* = 1912.1 (2) Å^3^
*Z* = 4
Mo *K*α radiation
μ = 0.09 mm^−1^
*T* = 293 K
0.35 × 0.30 × 0.25 mm

### Data collection  

Bruker APEXII CCD diffractometer
Absorption correction: multi-scan (*SADABS*; Bruker, 2008 ▶) *T*_min_ = 0.971, *T*_max_ = 0.979
26372 measured reflections
3956 independent reflections
2814 reflections with *I* > 2σ(*I*)
*R*_int_ = 0.067

### Refinement  

*R*[*F* ^2^ > 2σ(*F* ^2^)] = 0.058
*wR*(*F* ^2^) = 0.172
*S* = 0.98
3956 reflections
262 parameters
H-atom parameters constrained
Δρ_max_ = 0.56 e Å^−3^
Δρ_min_ = −0.45 e Å^−3^

### 

Data collection: *APEX2* (Bruker, 2008 ▶); cell refinement: *SAINT* (Bruker, 2008 ▶); data reduction: *SAINT*; program(s) used to solve structure: *SHELXS97* (Sheldrick, 2008 ▶); program(s) used to refine structure: *SHELXL97* (Sheldrick, 2008 ▶); molecular graphics: *ORTEP-3 for Windows* (Farrugia, 2012 ▶); software used to prepare material for publication: *SHELXL97* and *PLATON* (Spek, 2009 ▶).

## Supplementary Material

Crystal structure: contains datablock(s) I, New_Global_Publ_Block. DOI: 10.1107/S1600536814020868/su2785sup1.cif

Structure factors: contains datablock(s) I. DOI: 10.1107/S1600536814020868/su2785Isup2.hkl

Supporting information file. DOI: 10.1107/S1600536814020868/su2785Isup3.cml

**Fig. 1.**
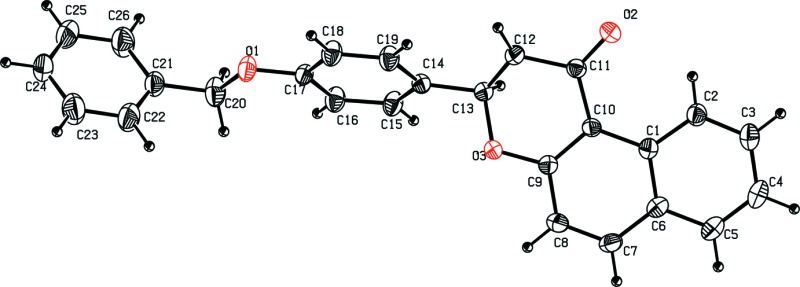
The molecular structure of the title molecule, with atom labelling. Displacement ellipsoids are drawn at the 30% probability level.

. DOI: 10.1107/S1600536814020868/su2785fig1.tif
The mol­ecular structure of the title mol­ecule, with atom labelling. Displacement ellipsoids are drawn at the 30% probability level.

**Fig. 2.**
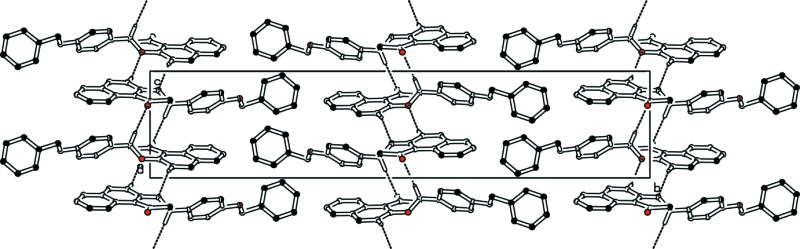
The crystal packing viewed along the a axis of the title compound. The dashed lines indicate the hydrogen bonds (see Table 1 for details).

. DOI: 10.1107/S1600536814020868/su2785fig2.tif
The crystal packing viewed along the a axis of the title compound. The dashed lines indicate the hydrogen bonds (see Table 1 for details).

CCDC reference: 1024680

Additional supporting information:  crystallographic information; 3D view; checkCIF report

## Figures and Tables

**Table 1:** Hydrogen-bond geometry (Å, °) *Cg*2 and *Cg*4 are the centroids of rings C1–C6 and C14–C19, respectively.

*D*—H⋯*A*	*D*—H	H⋯*A*	*D*⋯*A*	*D*—H⋯*A*
C8—H8⋯O3^i^	0.93	2.50	3.342 (3)	150
C13—H13⋯O2^ii^	0.98	2.51	3.314 (3)	140
C7—H7⋯*Cg*4^i^	0.93	2.96	3.688 (2)	136
C16—H16⋯*Cg*2^iii^	0.93	2.90	3.602 (2)	133
C19—H19⋯*Cg*2^iv^	0.93	2.97	3.593 (2)	126

Symmetry codes: (i) 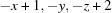; (ii) 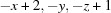; (iii) 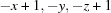; (iv) 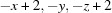.

## supplementary crystallographic information

### S1. Experimental

The title compound was synthesized following the reported procedure (Kumar *et al.*, 2014). The crude product produced was recrystallized twice from chloroform yielding block-like colourless crystals.

### S2. Refinement

H atoms were positioned geometrically and treated as riding on their parent atoms, with C—H distance of 0.93–0.98 Å, with *U*_iso_(H)= 1.5 *U*_eq_(C-methyl) and *U*_iso_(H)= 1.2Ueq(C) for other H atom.

### Crystal data

**Table:**

C_26_H_19_O_3_	*Z* = 4
*M**_r_* = 379.41	*F*(000) = 796.0
Monoclinic, *P*2_1_/*n*	*D*_x_ = 1.318 Mg m^−^^3^
Hall symbol: -P 2yn	Mo *K*α radiation, λ = 0.71073 Å
*a* = 6.9632 (4) Å	Cell parameters from 3956 reflections
*b* = 35.846 (2) Å	µ = 0.09 mm^−^^1^
*c* = 7.7879 (5) Å	*T* = 293 K
β = 100.375 (3)°	Block, colourless
*V* = 1912.1 (2) Å^3^	0.35 × 0.30 × 0.25 mm

### Data collection

**Table:**

Bruker APEXII CCD diffractometer	3956 independent reflections
Radiation source: fine-focus sealed tube	2814 reflections with *I* > 2σ(*I*)
Graphite monochromator	*R*_int_ = 0.067
ω and φ scan	θ_max_ = 26.5°, θ_min_ = 1.1°
Absorption correction: multi-scan (*SADABS*; Bruker, 2008)	*h* = −8→8
*T*_min_ = 0.971, *T*_max_ = 0.979	*k* = −44→44
26372 measured reflections	*l* = −8→9

### Refinement

**Table:**

Refinement on *F*^2^	Primary atom site location: structure-invariant direct methods
Least-squares matrix: full	Secondary atom site location: difference Fourier map
*R*[*F*^2^ > 2σ(*F*^2^)] = 0.058	Hydrogen site location: inferred from neighbouring sites
*wR*(*F*^2^) = 0.172	H-atom parameters constrained
*S* = 0.98	*w* = 1/[σ^2^(*F*_o_^2^) + (0.0897*P*)^2^ + 0.9692*P*] where *P* = (*F*_o_^2^ + 2*F*_c_^2^)/3
3956 reflections	(Δ/σ)_max_ < 0.001
262 parameters	Δρ_max_ = 0.56 e Å^−^^3^
0 restraints	Δρ_min_ = −0.45 e Å^−^^3^

### Special details

**Table:**

Geometry. All e.s.d.'s (except the e.s.d. in the dihedral angle between two l.s. planes) are estimated using the full covariance matrix. The cell e.s.d.'s are taken into account individually in the estimation of e.s.d.'s in distances, angles and torsion angles; correlations between e.s.d.'s in cell parameters are only used when they are defined by crystal symmetry. An approximate (isotropic) treatment of cell e.s.d.'s is used for estimating e.s.d.'s involving l.s. planes.
Refinement. Refinement of *F*^2^ against ALL reflections. The weighted *R*-factor *wR* and goodness of fit *S* are based on *F*^2^, conventional *R*-factors *R* are based on *F*, with *F* set to zero for negative *F*^2^. The threshold expression of *F*^2^ > σ(*F*^2^) is used only for calculating *R*-factors(gt) *etc*. and is not relevant to the choice of reflections for refinement. *R*-factors based on *F*^2^ are statistically about twice as large as those based on *F*, and *R*- factors based on ALL data will be even larger.

### Fractional atomic coordinates and isotropic or equivalent isotropic displacement parameters (Å^2^)

**Table:**

	*x*	*y*	*z*	*U*_iso_*/*U*_eq_	
C22	0.0790 (5)	0.22907 (8)	0.9025 (5)	0.0828 (10)	
H22	0.0911	0.2093	0.9810	0.099*	
C23	0.0400 (6)	0.26438 (9)	0.9588 (5)	0.0856 (10)	
H23	0.0287	0.2683	1.0746	0.103*	
C25	0.0388 (5)	0.28758 (8)	0.6783 (5)	0.0820 (10)	
H25	0.0254	0.3075	0.6004	0.098*	
C1	0.9499 (3)	−0.06874 (5)	0.7729 (3)	0.0373 (5)	
C2	1.1154 (3)	−0.08336 (6)	0.7143 (3)	0.0450 (5)	
H2	1.2012	−0.0671	0.6736	0.054*	
C3	1.1523 (4)	−0.12073 (7)	0.7161 (3)	0.0543 (6)	
H3	1.2610	−0.1295	0.6746	0.065*	
C4	1.0296 (4)	−0.14605 (6)	0.7791 (3)	0.0568 (7)	
H4	1.0565	−0.1715	0.7800	0.068*	
C5	0.8703 (4)	−0.13318 (6)	0.8392 (3)	0.0514 (6)	
H5	0.7891	−0.1500	0.8822	0.062*	
C6	0.8258 (3)	−0.09490 (6)	0.8376 (3)	0.0412 (5)	
C7	0.6594 (3)	−0.08175 (6)	0.8992 (3)	0.0482 (6)	
H7	0.5810	−0.0987	0.9451	0.058*	
C8	0.6111 (3)	−0.04520 (6)	0.8932 (3)	0.0465 (5)	
H8	0.5002	−0.0373	0.9338	0.056*	
C9	0.7292 (3)	−0.01921 (6)	0.8251 (3)	0.0375 (5)	
C10	0.8997 (3)	−0.02972 (5)	0.7698 (3)	0.0344 (4)	
C11	1.0247 (3)	0.00040 (6)	0.7191 (3)	0.0364 (5)	
C12	0.9490 (3)	0.03946 (6)	0.7283 (3)	0.0408 (5)	
H12	1.0277	0.0604	0.7525	0.049*	
C13	0.7290 (3)	0.04024 (5)	0.6933 (3)	0.0373 (5)	
H13	0.6829	0.0297	0.5770	0.045*	
C14	0.6330 (3)	0.07754 (5)	0.7027 (3)	0.0371 (5)	
C15	0.4393 (3)	0.08196 (6)	0.6255 (3)	0.0481 (6)	
H15	0.3734	0.0620	0.5656	0.058*	
C16	0.3404 (3)	0.11518 (6)	0.6346 (3)	0.0498 (6)	
H16	0.2101	0.1173	0.5815	0.060*	
C17	0.4364 (3)	0.14510 (5)	0.7228 (3)	0.0400 (5)	
C18	0.6314 (3)	0.14146 (6)	0.7982 (3)	0.0507 (6)	
H18	0.6982	0.1617	0.8553	0.061*	
C19	0.7269 (3)	0.10812 (6)	0.7892 (3)	0.0492 (6)	
H19	0.8573	0.1060	0.8422	0.059*	
C20	0.1518 (4)	0.18447 (7)	0.6774 (4)	0.0630 (7)	
H20A	0.0746	0.1657	0.7234	0.076*	
H20B	0.1266	0.1827	0.5510	0.076*	
C21	0.1004 (3)	0.22258 (6)	0.7340 (4)	0.0529 (6)	
C24	0.0182 (4)	0.29332 (7)	0.8453 (5)	0.0685 (8)	
H24	−0.0108	0.3170	0.8822	0.082*	
C26	0.0797 (5)	0.25237 (8)	0.6224 (4)	0.0744 (9)	
H26	0.0935	0.2489	0.5070	0.089*	
O1	0.3549 (2)	0.17907 (4)	0.7444 (2)	0.0519 (4)	
O2	1.1831 (2)	−0.00482 (4)	0.6780 (2)	0.0514 (4)	
O3	0.6623 (2)	0.01632 (4)	0.8212 (2)	0.0444 (4)	

### Atomic displacement parameters (Å^2^)

**Table:**

	*U*^11^	*U*^22^	*U*^33^	*U*^12^	*U*^13^	*U*^23^
C22	0.116 (3)	0.0570 (18)	0.085 (2)	0.0332 (17)	0.042 (2)	0.0208 (15)
C23	0.116 (3)	0.072 (2)	0.076 (2)	0.0312 (19)	0.036 (2)	0.0007 (16)
C25	0.106 (3)	0.0529 (17)	0.093 (3)	0.0263 (16)	0.034 (2)	0.0219 (16)
C1	0.0407 (11)	0.0366 (11)	0.0335 (11)	0.0028 (8)	0.0035 (9)	−0.0002 (8)
C2	0.0477 (13)	0.0410 (12)	0.0466 (13)	0.0067 (9)	0.0088 (10)	0.0024 (10)
C3	0.0565 (14)	0.0479 (14)	0.0575 (15)	0.0153 (11)	0.0076 (12)	−0.0022 (11)
C4	0.0704 (17)	0.0349 (12)	0.0602 (16)	0.0083 (11)	−0.0015 (13)	−0.0017 (11)
C5	0.0586 (15)	0.0365 (12)	0.0555 (15)	−0.0049 (10)	0.0009 (12)	0.0033 (10)
C6	0.0447 (12)	0.0372 (11)	0.0396 (12)	−0.0039 (9)	0.0016 (9)	0.0034 (9)
C7	0.0489 (13)	0.0425 (13)	0.0553 (14)	−0.0072 (10)	0.0148 (11)	0.0086 (10)
C8	0.0433 (12)	0.0494 (13)	0.0506 (13)	0.0003 (10)	0.0183 (10)	0.0058 (10)
C9	0.0409 (11)	0.0353 (11)	0.0378 (11)	0.0007 (8)	0.0108 (9)	0.0011 (8)
C10	0.0369 (10)	0.0356 (10)	0.0313 (10)	0.0016 (8)	0.0079 (8)	0.0023 (8)
C11	0.0360 (11)	0.0389 (11)	0.0347 (11)	0.0008 (8)	0.0077 (8)	0.0019 (8)
C12	0.0389 (11)	0.0337 (11)	0.0507 (13)	−0.0011 (8)	0.0109 (9)	0.0012 (9)
C13	0.0411 (11)	0.0344 (11)	0.0379 (11)	0.0008 (8)	0.0110 (9)	0.0015 (8)
C14	0.0396 (11)	0.0359 (11)	0.0371 (11)	0.0028 (8)	0.0099 (9)	0.0001 (8)
C15	0.0471 (13)	0.0356 (12)	0.0585 (15)	0.0005 (9)	0.0010 (11)	−0.0072 (10)
C16	0.0422 (12)	0.0426 (13)	0.0608 (15)	0.0060 (9)	−0.0008 (11)	−0.0040 (11)
C17	0.0469 (12)	0.0311 (10)	0.0430 (12)	0.0051 (9)	0.0106 (10)	0.0023 (8)
C18	0.0493 (13)	0.0392 (12)	0.0602 (15)	−0.0005 (10)	0.0011 (11)	−0.0102 (10)
C19	0.0400 (12)	0.0430 (13)	0.0615 (16)	0.0045 (9)	0.0010 (10)	−0.0060 (10)
C20	0.0506 (14)	0.0508 (15)	0.083 (2)	0.0120 (11)	0.0008 (13)	−0.0111 (13)
C21	0.0452 (13)	0.0426 (13)	0.0702 (17)	0.0091 (10)	0.0090 (12)	−0.0011 (11)
C24	0.0710 (18)	0.0450 (15)	0.093 (2)	0.0124 (12)	0.0234 (16)	−0.0042 (14)
C26	0.094 (2)	0.0689 (19)	0.0622 (18)	0.0301 (16)	0.0193 (16)	0.0089 (14)
O1	0.0497 (9)	0.0351 (8)	0.0694 (11)	0.0092 (7)	0.0063 (8)	−0.0039 (7)
O2	0.0432 (9)	0.0484 (9)	0.0678 (11)	0.0025 (7)	0.0236 (8)	0.0018 (8)
O3	0.0485 (9)	0.0363 (8)	0.0545 (10)	0.0070 (6)	0.0256 (7)	0.0050 (7)

### Geometric parameters (Å, º)

**Table:**

C22—C21	1.367 (4)	C10—C11	1.484 (3)
C22—C23	1.383 (4)	C11—O2	1.218 (2)
C22—H22	0.9300	C11—C12	1.502 (3)
C23—C24	1.353 (4)	C12—C13	1.507 (3)
C23—H23	0.9300	C12—H12	0.9300
C25—C24	1.350 (5)	C13—O3	1.453 (2)
C25—C26	1.381 (4)	C13—C14	1.503 (3)
C25—H25	0.9300	C13—H13	0.9800
C1—C2	1.414 (3)	C14—C15	1.384 (3)
C1—C6	1.428 (3)	C14—C19	1.387 (3)
C1—C10	1.441 (3)	C15—C16	1.384 (3)
C2—C3	1.364 (3)	C15—H15	0.9300
C2—H2	0.9300	C16—C17	1.380 (3)
C3—C4	1.394 (4)	C16—H16	0.9300
C3—H3	0.9300	C17—O1	1.367 (2)
C4—C5	1.360 (4)	C17—C18	1.385 (3)
C4—H4	0.9300	C18—C19	1.376 (3)
C5—C6	1.406 (3)	C18—H18	0.9300
C5—H5	0.9300	C19—H19	0.9300
C6—C7	1.412 (3)	C20—O1	1.430 (3)
C7—C8	1.352 (3)	C20—C21	1.499 (3)
C7—H7	0.9300	C20—H20A	0.9700
C8—C9	1.408 (3)	C20—H20B	0.9700
C8—H8	0.9300	C21—C26	1.368 (4)
C9—O3	1.355 (2)	C24—H24	0.9300
C9—C10	1.386 (3)	C26—H26	0.9300
			
C21—C22—C23	121.5 (3)	C11—C12—H12	124.4
C21—C22—H22	119.3	C13—C12—H12	124.4
C23—C22—H22	119.3	O3—C13—C14	106.96 (15)
C24—C23—C22	120.0 (3)	O3—C13—C12	107.76 (16)
C24—C23—H23	120.0	C14—C13—C12	117.02 (17)
C22—C23—H23	120.0	O3—C13—H13	108.3
C24—C25—C26	120.4 (3)	C14—C13—H13	108.3
C24—C25—H25	119.8	C12—C13—H13	108.3
C26—C25—H25	119.8	C15—C14—C19	117.26 (19)
C2—C1—C6	116.79 (19)	C15—C14—C13	119.22 (18)
C2—C1—C10	124.30 (19)	C19—C14—C13	123.50 (19)
C6—C1—C10	118.91 (19)	C14—C15—C16	122.1 (2)
C3—C2—C1	121.6 (2)	C14—C15—H15	118.9
C3—C2—H2	119.2	C16—C15—H15	118.9
C1—C2—H2	119.2	C17—C16—C15	119.6 (2)
C2—C3—C4	121.1 (2)	C17—C16—H16	120.2
C2—C3—H3	119.4	C15—C16—H16	120.2
C4—C3—H3	119.4	O1—C17—C16	125.4 (2)
C5—C4—C3	119.3 (2)	O1—C17—C18	115.40 (19)
C5—C4—H4	120.3	C16—C17—C18	119.21 (19)
C3—C4—H4	120.3	C19—C18—C17	120.4 (2)
C4—C5—C6	121.3 (2)	C19—C18—H18	119.8
C4—C5—H5	119.3	C17—C18—H18	119.8
C6—C5—H5	119.3	C18—C19—C14	121.4 (2)
C5—C6—C7	121.0 (2)	C18—C19—H19	119.3
C5—C6—C1	119.8 (2)	C14—C19—H19	119.3
C7—C6—C1	119.11 (19)	O1—C20—C21	106.8 (2)
C8—C7—C6	121.8 (2)	O1—C20—H20A	110.4
C8—C7—H7	119.1	C21—C20—H20A	110.4
C6—C7—H7	119.1	O1—C20—H20B	110.4
C7—C8—C9	119.7 (2)	C21—C20—H20B	110.4
C7—C8—H8	120.1	H20A—C20—H20B	108.6
C9—C8—H8	120.1	C26—C21—C22	117.4 (2)
O3—C9—C10	123.98 (18)	C26—C21—C20	121.9 (3)
O3—C9—C8	114.13 (18)	C22—C21—C20	120.7 (2)
C10—C9—C8	121.89 (19)	C23—C24—C25	119.6 (3)
C9—C10—C1	118.51 (18)	C23—C24—H24	120.2
C9—C10—C11	117.45 (17)	C25—C24—H24	120.2
C1—C10—C11	123.97 (18)	C21—C26—C25	121.1 (3)
O2—C11—C10	124.10 (18)	C21—C26—H26	119.4
O2—C11—C12	119.87 (18)	C25—C26—H26	119.4
C10—C11—C12	115.97 (17)	C17—O1—C20	118.75 (18)
C11—C12—C13	111.26 (17)	C9—O3—C13	114.78 (15)
			
C21—C22—C23—C24	1.3 (6)	C11—C12—C13—C14	178.52 (17)
C6—C1—C2—C3	1.4 (3)	O3—C13—C14—C15	−77.2 (2)
C10—C1—C2—C3	−178.2 (2)	C12—C13—C14—C15	161.9 (2)
C1—C2—C3—C4	−1.2 (4)	O3—C13—C14—C19	101.1 (2)
C2—C3—C4—C5	0.2 (4)	C12—C13—C14—C19	−19.8 (3)
C3—C4—C5—C6	0.6 (4)	C19—C14—C15—C16	−0.7 (4)
C4—C5—C6—C7	179.6 (2)	C13—C14—C15—C16	177.7 (2)
C4—C5—C6—C1	−0.4 (3)	C14—C15—C16—C17	0.1 (4)
C2—C1—C6—C5	−0.6 (3)	C15—C16—C17—O1	−178.6 (2)
C10—C1—C6—C5	178.97 (19)	C15—C16—C17—C18	1.1 (4)
C2—C1—C6—C7	179.5 (2)	O1—C17—C18—C19	178.1 (2)
C10—C1—C6—C7	−1.0 (3)	C16—C17—C18—C19	−1.7 (4)
C5—C6—C7—C8	−177.8 (2)	C17—C18—C19—C14	1.1 (4)
C1—C6—C7—C8	2.2 (3)	C15—C14—C19—C18	0.1 (4)
C6—C7—C8—C9	−0.4 (4)	C13—C14—C19—C18	−178.2 (2)
C7—C8—C9—O3	178.1 (2)	C23—C22—C21—C26	−0.6 (5)
C7—C8—C9—C10	−2.7 (3)	C23—C22—C21—C20	177.2 (3)
O3—C9—C10—C1	−177.06 (19)	O1—C20—C21—C26	102.0 (3)
C8—C9—C10—C1	3.8 (3)	O1—C20—C21—C22	−75.7 (3)
O3—C9—C10—C11	5.9 (3)	C22—C23—C24—C25	−1.3 (6)
C8—C9—C10—C11	−173.2 (2)	C26—C25—C24—C23	0.7 (5)
C2—C1—C10—C9	177.6 (2)	C22—C21—C26—C25	0.0 (5)
C6—C1—C10—C9	−1.9 (3)	C20—C21—C26—C25	−177.8 (3)
C2—C1—C10—C11	−5.6 (3)	C24—C25—C26—C21	0.0 (5)
C6—C1—C10—C11	174.91 (19)	C16—C17—O1—C20	2.6 (3)
C9—C10—C11—O2	175.6 (2)	C18—C17—O1—C20	−177.1 (2)
C1—C10—C11—O2	−1.3 (3)	C21—C20—O1—C17	175.0 (2)
C9—C10—C11—C12	−1.8 (3)	C10—C9—O3—C13	24.6 (3)
C1—C10—C11—C12	−178.61 (19)	C8—C9—O3—C13	−156.19 (19)
O2—C11—C12—C13	152.1 (2)	C14—C13—O3—C9	177.39 (17)
C10—C11—C12—C13	−30.4 (2)	C12—C13—O3—C9	−56.0 (2)
C11—C12—C13—O3	58.0 (2)		

### Hydrogen-bond geometry (Å, º)

Cg2 and Cg4 are the centroids of rings C1–C6 and C14–C19, respectively.

**Table:**

*D*—H···*A*	*D*—H	H···*A*	*D*···*A*	*D*—H···*A*
C8—H8···O3^i^	0.93	2.50	3.342 (3)	150
C13—H13···O2^ii^	0.98	2.51	3.314 (3)	140
C7—H7···*Cg*4^i^	0.93	2.96	3.688 (2)	136
C16—H16···*Cg*2^iii^	0.93	2.90	3.602 (2)	133
C19—H19···*Cg*2^iv^	0.93	2.97	3.593 (2)	126

Symmetry codes: (i) −*x*+1, −*y*, −*z*+2; (ii) −*x*+2, −*y*, −*z*+1; (iii) −*x*+1, −*y*, −*z*+1; (iv) −*x*+2, −*y*, −*z*+2.
