# Supplementary figures and images for: Crystal structure of 3-[4-(benz­yloxy)phen­yl]-2,3-di­hydro-1H-benzo[f]chromen-1-one
Source: Acta Crystallogr Sect E Struct Rep Online. 2014 Sep 20;70(Pt 10):o1116–7. doi: 10.1107/S1600536814020868 (PMC4257196; doi:10.1107/S1600536814020868)

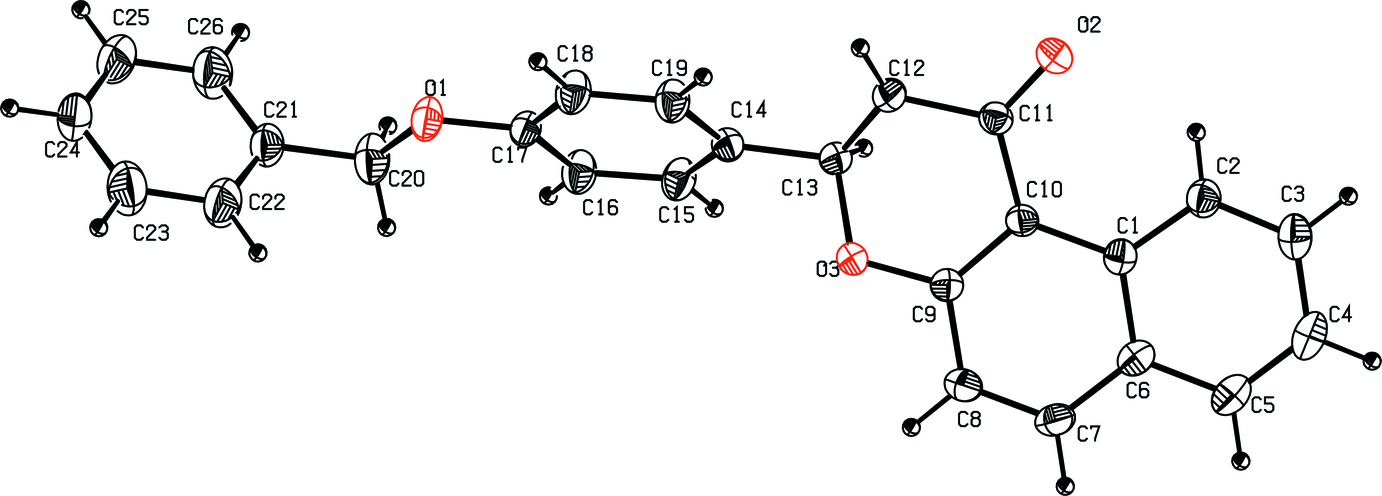

Supplement: Supplementary file 4 [file e-70-o1116-fig1.tif]

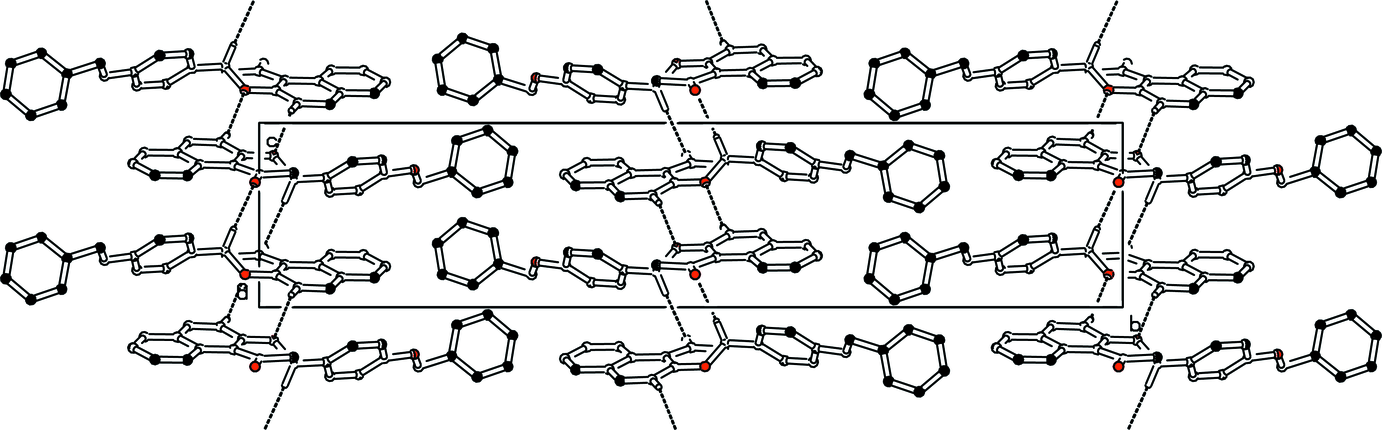

Supplement: Supplementary file 5 [file e-70-o1116-fig2.tif]
